# Site-Directed Spin Labeling of RNA with a *Gem*-Diethylisoindoline Spin Label: PELDOR, Relaxation, and Reduction Stability

**DOI:** 10.3390/molecules24244482

**Published:** 2019-12-06

**Authors:** Christine Wuebben, Simon Blume, Dinar Abdullin, Dominik Brajtenbach, Florian Haege, Stephanie Kath-Schorr, Olav Schiemann

**Affiliations:** 1 Institute of Physical and Theoretical Chemistry, University of Bonn, Wegelerstraße 12, 53115 Bonn, Germany; wuebben@pc.uni-bonn.de (C.W.); s6siblum@uni-bonn.de (S.B.); abdullin@pc.uni-bonn.de (D.A.); s6dobraj@uni-bonn.de (D.B.); Florian-Haege@web.de (F.H.); 2 Life & Medical Sciences Institute Chemical Biology & Medicinal Chemistry Unit, University of Bonn, Gerhard-Domagk-Straße 1, 53121 Bonn, Germany; stephanie.kath-schorr@uni-bonn.de

**Keywords:** EPR, spin labeling, nitroxide, PELDOR, RNA, relaxation time, in-cell

## Abstract

Ribonucleic acid function is governed by its structure, dynamics, and interaction with other biomolecules and influenced by the local environment. Thus, methods are needed that enable one to study RNA under conditions as natural as possible, possibly within cells. Site-directed spin-labeling of RNA with nitroxides in combination with, for example, pulsed electron–electron double resonance (PELDOR or DEER) spectroscopy has been shown to provide such information. However, for in-cell measurements, the usually used *gem*-dimethyl nitroxides are less suited, because they are quickly reduced under in-cell conditions. In contrast, *gem*-diethyl nitroxides turned out to be more stable, but labeling protocols for binding these to RNA have been sparsely reported. Therefore, we describe here the bioconjugation of an azide functionalized *gem*-diethyl isoindoline nitroxide to RNA using a copper (I)-catalyzed azide–alkyne cycloaddition (“click”-chemistry). The labeling protocol provides high yields and site selectivity. The analysis of the orientation selective PELDOR data show that the *gem*-diethyl and *gem*-dimethyl labels adopt similar conformations. Interestingly, in deuterated buffer, both labels attached to RNA yield *T_M_* relaxation times that are considerably longer than observed for the same type of label attached to proteins, enabling PELDOR time windows of up to 20 microseconds. Together with the increased stability in reducing environments, this label is very promising for in-cell Electron Paramagnetic Resonance (EPR) studies.

## 1. Introduction

**Figure 1:**
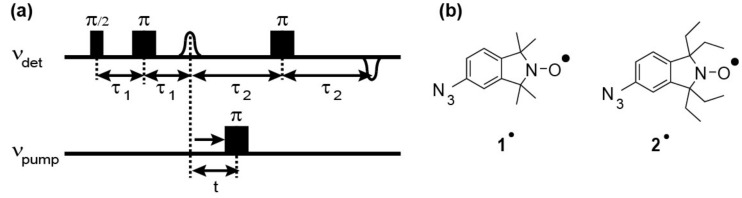
(**a**) Scheme of the pulsed electron–electron double resonance (PELDOR) pulse sequence and (**b**) Lewis structures of spin labels **1**^•^ and **2**^•^.

Ribonucleic acid function is usually associated with the translation of the genetic code into proteins involving mRNAs, tRNAs, and rRNAs. However, over the last decades, it was found that RNA fulfills many more roles. For example, snRNAs (small nuclear RNA) function in a variety of nuclear processes including the splicing of pre-mRNA [1]; snoRNAs (small nucleolar RNA) process and chemically modify rRNA [2]; ribozymes are catalytically active RNAs [3]; and riboswitches [4] play an important role in the regulation of genes. In order to understand how RNA is able to perform this wide range of functions, insight into its three-dimensional fold, its conformational dynamics, and complex formation with other biomolecules is required. Through the years, a toolbox of biophysical methods by which such insights can be obtained has been assembled. These methods include X-ray crystallography [5,6], cryo-electron microscopy (cryoEM) [7,8], nuclear magnetic resonance spectroscopy (NMR) [9,10], electron paramagnetic resonance (EPR) [11,12], Förster resonance energy transfer (FRET) [13,14], small-angle X-ray scattering (SAXS) [15], and molecular dynamics simulation (MD) [16]. Out of this toolbox, EPR is a method that provides such information but requires the presence of unpaired electrons. Since RNA is intrinsically diamagnetic, the spin centers have to be introduced, for example, by means of a diamagnetic for paramagnetic metal ion exchange (e.g., Mg(II) for Mn(II) [17,18]) or by site-directed spin labeling (SDSL) with nitroxides [19,20], trityl radicals [21,22,23] or metal complexes based on, for example, gadolinium [24,25]. Over the years, various methods have been established to attach these labels site specifically to the sugar [26,27], phosphate [28,29,30] or base moiety of RNA [31,32,33,34,35,36]. The conjugation can be performed either during the oligonucleotide synthesis or through post-synthetic labeling of pre-functionalized sites of the oligonucleotide. If two or more spin centers are present, EPR-based pulsed dipolar spectroscopy (PDS) offers a means to measure the distance between these centers up to 16 nm [37] and thereby gain structural and dynamics information. Out of the PDS methods, pulsed electron–electron double resonance (PELDOR or DEER, Figure 1) in combination with nitroxide spin labeling has been particularly successful in determining biomolecular structures in solutions to monitor their conformational changes and to access their internal dynamics [38,39]. However, in order to apply SDSL/PELDOR within cells, the spin labels have to survive reduction in cell conditions. In this regard, the usually used *gem*-dimethyl nitroxides have the disadvantage of being reduced within minutes. In contrast, *gem*-diethyl nitroxide or nitroxides with sterically even more demanding groups in the *gem*-position reveal increased redox stability [27,40,41,42,43,44,45,46]. Yet, protocols for spin-labeling DNA [47] or RNA [27] with *gem*-diethyl isoindoline nitroxides have been reported only rarely. Previously, we reported on the bioconjugation of the *gem*-dimethyl nitroxide **1^•^** (Figure 1b) to RNA using “click”-chemistry in solution [48].

Here, this protocol was extended to the azide functionalized *gem*-diethylisoindoline spin label **2**^•^ and its stability under reducing conditions, its relaxation behavior and performance in PELDOR measurements were tested.

## 2. Results and Discussion

**Figure 2:**
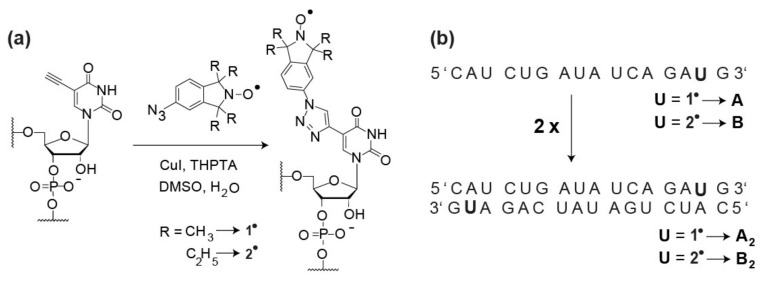
(**a**) Spin labeling reaction based on the “click”-reaction. THPTA = Tris(3-hydroxypropyltriazolyl-methyl)amine; DMSO = dimethylsulfoxide. (**b**) Sequence of the single-stranded RNA and the annealed duplex. The letter **U** marked in bold corresponds to the labeling positions. In the following, the RNA single strand labeled with **1**^•^ is called **A** while the one labeled with **2**^•^ is called **B**. The spin labeled single-stranded RNAs **A** and **B** were annealed to obtain duplexes **A_2_** and **B_2_**, respectively.

**Figure 3:**
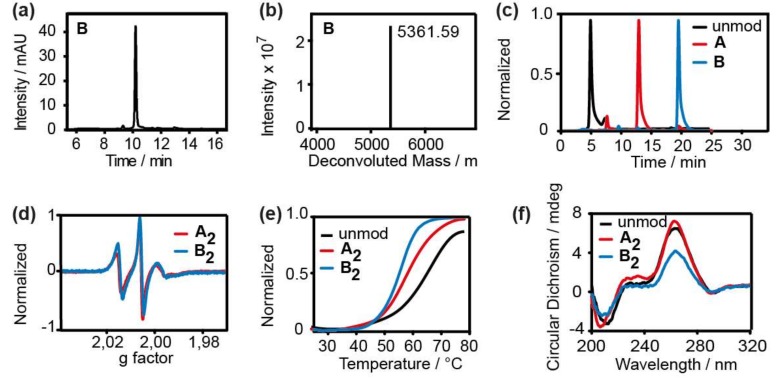
(**a**) LCMS-UV Chromatogram at 260 nm and (**b**) deconvoluted mass of **B** (calculated mass 5361.03, found mass 5361.59). (**c**) Overlay of the HPLC runs of the unmodified (black line) and labeled RNAs **A** (red line) and **B** (blue line). (**d**) Experimental cw EPR spectra of 35 µM **A_2_** (red line) and 35 µM **B_2_** (blue line). (**e**) Thermal denaturation curves of the unmodified (black line, T_m_ = 66 °C) and labeled RNAs **A_2_** (red line, T_m_ = 58 °C) and **B_2_** (blue line, T_m_ = 55 °C). (**f**) Circular Dichroism (CD) spectra of the unmodified (black line) and labeled RNAs **A_2_** (red line) and **B_2_** (blue line).

Previously, we showed that the *gem*-dimethylisoindoline label **1**^•^ can be attached to RNA strands in solution employing the CuAAC “click”-reaction (Figure 2a) [48]. Here, this protocol was extended to the *gem*-diethylisoindoline analogue **2**^•^ (see Supplementary Materials for synthesis), using the same self-complementary RNA strand (Figure 2b) as for **1**^•^ [34]. The reaction was carried out by first preparing the catalytic copper (I) solution and then incubating it with the alkyne-modified RNA and the spin label at 60 °C for 30 min. Subsequently, the labelled RNA was purified by means of HPLC. The yield of labelled RNA was 50% with respect to starting RNA. Incorporation of the label as well as the purity of the sample was confirmed by LC-MS (Figure 3a–c). The liquid state cw X-band EPR spectrum of **B_2_** is depicted in Figure 3d and shows the typical line-shape of immobilized nitroxides, very similar to the spectrum of **A_2_** [34]. Spin counting revealed that all purified RNA strands were quantitatively labelled (Table S1). The same protocol also works for DNA as shown in Supplementary Materials Section S6.2.

Since every modification of RNA potentially may result in structural changes, the influence of the spin label on the duplex structure was evaluated using UV-Vis-based melting studies (Figure 3e) and Circular Dichroism (CD) spectroscopy (Figure 3f). For **B_2_**, a decrease in the melting temperature T_m_ of 5.5 °C per spin label was found which is in the usual range [31] and similar to the decrease of 4 °C found previously for **A_2_ [34]**. Also, the CD spectra showed only minor changes in the amplitudes confirming the prevalence of the standard A-form of the duplex. Thus, the influence of **2**^•^ on the RNA duplex structure was judged as being minor and relating only to local structural perturbations.

### 2.1. Two-Pulse Electron Spin Echo Envelope Modulation (ESEEM) Measurements

**Figure 4:**
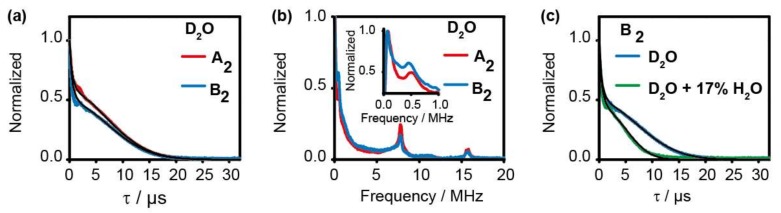
(**a**) Two-pulse Electron Spin Echo Envelope Modulation (ESEEM) decay curves of **A_2_** (red line) and **B_2_** (blue line) in deuterated buffer at 50 K. (**b**) Frequency domain spectrum of the time traces in (**a**) for **A_2_** (red line) and **B_2_** (blue line). The inset is a zoom-in for the frequency range 0–1 MHz. (**c**) Two-pulse ESEEM echo decay curves of **B_2_** in pure deuterated buffer (blue line) and in deuterated buffer with 17% additional water (green line) both at 50 K.

The phase memory time, *T*_M_, of the spin label attached to the biomolecule is interesting for PELDOR measurements, because it determines the length of the PELDOR time trace and, thus, the distance length as well as the accuracy that can be accessed. Therefore, two-pulse ESEEM time traces were recorded at the Q-band for both RNAs, **A_2_** and **B_2_**, in deuterated phosphate buffer (145 mM sodium chloride, 10 mM sodium phosphate, pH 7.0 in D_2_O, 20% d6-ethyleneglycol) (Figure 4a). The echo-decays were fitted according to a stretched, biexponential decay:

(1) $$Y\left(2\tau \right)=\;Y_{0}\times \exp{\left(-2\tau /T_{M1}\right)}^{x1}+\left(1-Y_{0}\right)\times exp{\left(-2\tau /T_{M2}\right)}^{x2}$$

Here, Y (2τ) is the intensity of the echo as a function of the time between the two pulses τ. *Y*_0_ is the echo intensity extrapolated to time zero. *T*_M1_ and *T*_M2_ are the phase memory times, and *x*1 and *x*2 are the corresponding stretched parameters, respectively. This expression contains 2τ in the exponent, since the relaxation process is effective during both periods of τ of the Hahn-echo sequence. A stretched bi-exponential decay was chosen according to the literature [49] and because it yielded the best fit (Figure S3). In the literature, the stretch parameters vary between *x* = 1, if the relaxation process is dominated by instantaneous spin diffusion, and *x* = 2 in the case of nuclei spin diffusion [50]. Often, as here, the relaxation is a function of both. Fitting the time traces yielded the parameters in Table 1 and indicates very similar transversal relaxation behavior for **A_2_** and **B_2_**. In both cases, the *T_M_* relaxation was dominated by a component with a time constant $T_{M1}$ of ~21 µs and a stretched exponent x1 of ~2, while the minor component was much faster with $T_{M2}$ of ~1 µs and x2 of ~1. Interestingly, the time constant of the dominating component decreased significantly to $T_{M1}$ of ~13 µs upon adding 17% H_2_O, while the minor component did not change (Figure 4c). This indicates that T_M1_ was dominated by nuclear spin diffusion [50]. That *T_M_* increased upon matrix deuteration was reported before [51,52], but labeling proteins with *gem*-diethylisoindoline or other nitroxides did not yield such long *T_M_* times. To our knowledge, such long *T_M_* times were only obtained if not only the solvent but also the biomolecule itself was deuterated [37,53,54]. For example, Ward et al. found similar *T_M_* values if the protein in the critical range of 7–25 Å from the spin center was deuterated [53,54,55,56]. Here, the length of label **2**^•^ placed the spin center at a minimum distance of 7–10 Å to the backbone of the RNA (Figure S7), and, in the case of oligonucleotides, many hydrogens are easily exchangeable by just diluting them in D_2_O which strongly increases the fraction of deuterium in the critical distance range. That this effect has not been reported previously for nitroxide-labeled oligonucleotides might be due to the particular length of the label and the thorough deuteration conditions used here.

In addition, the FFT-transformed ESEEM spectra showed a dipolar coupling frequency at 0.50 and 0.48 MHz for **A_2_** and **B_2_**, respectively (Figure 4b) which translate into distances of 4.7 and 4.8 nm, respectively. These distances are in good agreement with the PELDOR data (see below). The observation of dipolar coupling frequencies in two-pulse ESEEM spectra is rare but has been reported before by Ward et al. [53,54] and Kulik et al. [57].

### 2.2. PELDOR Measurements

**Figure 5:**
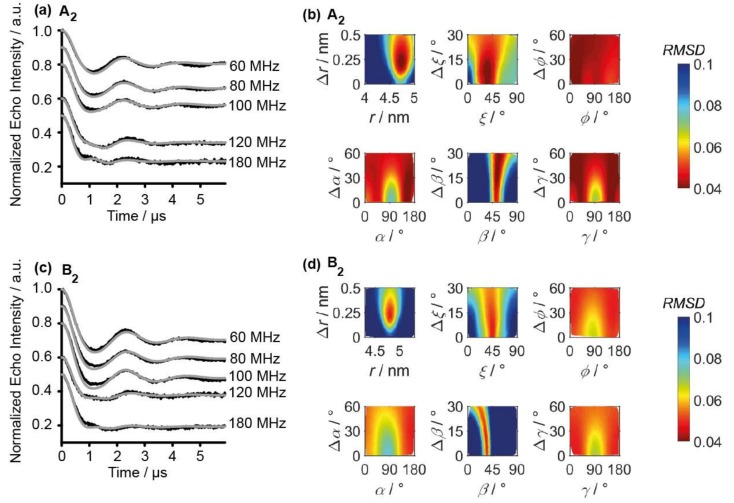
(**a**) Q-Band PELDOR time traces of **A_2_** acquired at different offsets (black lines) are overlaid with their PeldorFit simulations (grey lines). (**b**) RMSD between experimental and simulated PELDOR time traces as a function of the geometric parameter of PeldorFit for **A_2_**. (**c**) Q-Band PELDOR time traces of **B_2_** acquired at different offsets (black lines) are overlaid with their PeldorFit simulations (grey lines). (**d**) RMSD between experimental and simulated PELDOR time traces as a function of the geometric parameter of PeldorFit for **B_2_**.

Previously, it was shown that spin label **1**^•^ gives rise to orientation selection in PELDOR experiments [34] which is also seen here for **B_2_**, indicating the rigidity of the label at low temperatures [34]. Therefore, the analysis of the PELDOR data was performed with the program PeldorFit [58], taking orientation selectivity in account (see Supplementary Materials Section S5). The best fit to the time traces as well as the error plots for the distance and angular parameters are shown in Figure 5. The best values for each fitting parameter are collected in Table 2 and compared to the data of **A_2_**. The geometric parameters for **A_2_** and **B_2_** are very similar and are best defined for the distance *r* and the angles *ξ* and *β* (for definition see Figure S6). This indicates that, at least for duplex structures, the exchange methyl for ethyl in the *gem-*position does not induce more structural perturbations. This is also supported by the UV-Vis and CD study described above.

**Figure 6:**
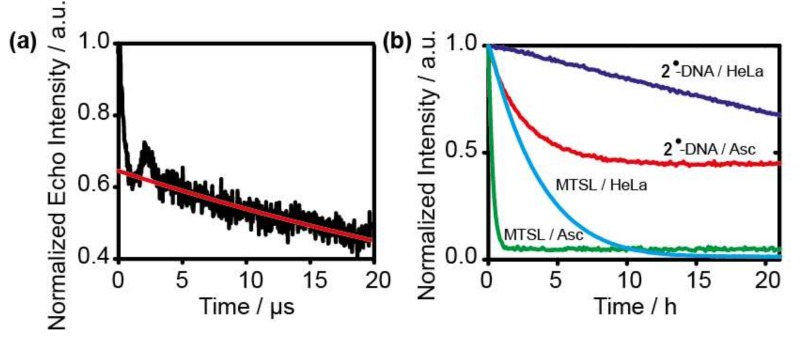
(**a**) Original PELDOR time trace of **B_2_** with an 80 MHz offsets in deuterated buffer at 50 K. (**b**) Lifetime of **2**^•^ attached to DNA and of MTSL in the presence of 4.75 mM ascorbate or in HeLa cell lysate (for more details see Supplementary Materials).

Based on the knowledge of the extended T_M_, it was also tested how long the PELDOR time window can be made while still achieving an acceptable signal-to-noise ratio within a measurement time of 24 h. Figure 6a shows that good data can be obtained up to 20 µs. Thus, distances up to 10 nm are achievable [56].

Finally, the stability of **2^•^** was accessed in two reducing media and compared to the *gem*-dimethyl analogue MTSL (Figure 6b). Due to the solubility of **2^•^**, it was attached to a DNA strand (see Supplementary Materials Section S6). It can be seen that MTSL decays rapidly within minutes in ascorbate, whereas **2^•^** is considerably more stable, prevailing for hours in the same medium. **2^•^** is also much more stable than **1^•^** in HeLa cell lysate, decaying only to 60% within 21 h.

## 3. Materials and Methods

### 3.1. General Procedures RNA

#### 3.1.1. RNA Sequence

The RNA with the sequence 5′ CAU CUG AUA UCA GAX G 3′ (X = U for non-modified RNA, X = 5-ethynyl-2′-dU for modified RNA) was purchased from metabion.

#### 3.1.2. Spin Labeling

First, 8 µL of a 250 mM solution of tris(3-hydroxypropyltriazolylmethyl)amine (THPTA, Sigma–Aldrich) in dimethylsulfoxide (DMSO, Carl Roth) were mixed with 8 µL of a fresh 50 mM solution of copper-I-iodide (Carl Roth) in DMSO. Then, 20 µL of DMSO were added and the mixture was incubated for 5 min at room temperature. Meanwhile, 2.5 nmol of the dried oligonucleotide with a 5-ethynyl-modified uridine at the desired position in the sequence was resuspended in 4.4 µL diethylpyrocarbonate (DEPC, Carl Roth) treated water. Then, 3.6 µL of the catalytic solution and 2 µL of 100 mM spin label solution in DMSO were added to the RNA solution. The reaction mixture was mixed well and incubated for 30 min at 60 °C and 300 rpm (Thermomixer comfort, Eppendorf, Hamburg, Germany). The reaction was quenched via adding 480 µL Milli-Q water and desalted via an Amicon© ultra 3K column (Merck). Afterwards, the RNA was purified through reverse-phase high-performance liquid chromatography with an Agilent 1200 Series HPLC System (Agilent Technology, Santa Clara, CA, USA) in combination with a Zorbax 300SB-C18 (4.6 mm × 150 mm) column (Agilent Technologies, Santa Clara, CA, USA). As the eluent was a 0.1 M aqueous solution of triethylammonium acetate (VWR Applichem), an increasing percentage of acetonitrile (VWR Chemicals) (8% to 20% over 20 min) was used. The fractions were desalted with Amicon Ultra-0.5 mL Centrifugal Filters (3K Device, Merck). Then, the RNA was either stored in Milli-Q water or rebuffered by washing the filter three times with 500 µL deuterated phosphate buffer (see Section 3.1.3.).

#### 3.1.3. EPR Sample Preparation

The RNA was annealed in phosphate buffer (145 mM sodium chloride (Carl Roth), 10 mM sodium phosphate (Acros Organics), pH 7.0) by heating to 70 °C for 1 min and then cooling to 5 °C with a rate of 1 °C/min. For the pulsed EPR measurements, the HPLC-purified RNA sample was rebuffered (see Section 3.1.2.) in deuterated phosphate buffer (D_2_O, Deutero) to yield RNA concentrations of 35 µM. Before freezing in liquid nitrogen, 20% d6-ethylene glycol was added (Sigma–Aldrich).

### 3.2. Chromatography

#### 3.2.1. HPLC

For the analysis of **2^•^**, Milli-Q water (Merck Millipore) was used with a gradient of 50% → 100% acetonitrile (Carl Roth) in 20 min on a Zorbax Eclipse XDB-C18 (4.6, 150 mm) column (Agilent Technologies, Santa Clara, CA, USA) with an Agilent 1200 Series HPLC System (Agilent Technology, Santa Clara, CA, USA).

#### 3.2.2. LCMS

For the analysis of the oligonucleotide, 10 mM trimethylamine (Fisher)/100 mM hexafluoroisoproanol (Fluka) was used with a gradient of 3% → 20% acetonitrile (VWR Chemicals) on a Zorbax Narrow Bore SB C18 (2.1 mm × 50 mm, 5 µm) column (Agilent Technology) in combination with an Agilent 1100 Series HPLC System (Agilent Technology, Santa Clara, CA, USA) and HTC esquire (Bruker Daltonik, Bremen, Germany).

### 3.3. Spectroscopy

#### 3.3.1. IR

The IR measurements were obtained from a Nicolet 5700 (Thermo Fisher, Waltham, MA, USA) FTIR spectrometer using a thin film of 150 µm of a 111 nM solution of **2^•^** in chloroform.

#### 3.3.2. CD

The CD spectra were recorded on a Jasco J-810 spectropolarimeter (Jasco, Tokyo, Japan) at room temperature with a scanning speed of 100 nm/min. To obtain a spectral range of 200–320 nm, a data pitch of 0.1 nm was adjusted. Ten scans were accumulated. The measurement was performed on 10 μM RNA samples.

#### 3.3.3. UV

The UV melting curves were recorded on a Cary 100 UV-Vis spectrophotometer (Agilent Technologies, Santa Clara, CA, USA) at a wavelength of 260 nm. The temperature of the sample was increased with a heating rate of 1 °C/min from 20 °C to 80 °C. The measurements were performed on 1 µM samples.

### 3.4. EPR

#### 3.4.1. cw EPR

The room temperature cw EPR spectrum of the 35 µM **A_2_** and **B_2_** were recorded at X-band frequencies on a Bruker EMXnano spectrometer (Bruker BioSpin, Rheinstetten, Germany) with a microwave power of 10 mW, a modulation frequency of 100 kHz, a modulation amplitude of 1.0 G, a microwave frequency of 9.641 GHz, and 1300 points in the field interval 337.9–350.9 mT.

#### 3.4.2. Pulsed EPR

The pulsed EPR measurements were conducted at Q-band frequencies on a Bruker ELEXSYS 580 EPR spectrometer (Bruker BioSpin, Rheinstetten, Germany) equipped with an ER 5106QT-II resonator and a 150 W TWT-amplifier (Applied System Engineering, Fort Worth, TX, USA). The temperature was adjusted to the appropriate value using a CF935 helium gas-flow cryostat (Oxford Instruments, Abingdon, UK) in conjugation with an Oxford Instruments ITC 502 temperature controller.

The ESEEM spectra were recorded using a standard two-pulse Hahn echo sequence with pulse lengths of 12 ns and 24 ns for the π and π/2 pulses, respectively, an initial τ of 200 ns, and with increments of τ of 8 ns.

The PELDOR experiments were performed at Q-band with the standard four-pulse sequence. The frequency of the pumping pulse was set at the maximum intensity of the nitroxide signal. The offset between pump and detection frequencies was varied as depicted in Figure S5 and Table S3. π/2 and π pulses lengths of 12 ns and 24 ns were used. For the π/2 pulse, a two-step phase cycle was executed. The pump pulse length was set to 12 ns. The initial τ was set to 260 ns (d1). Deuterium modulation was suppressed by addition of 8 time traces with an increment of 16 ns for τ. The detection window with a width of 40 ns was set to the maximum of the echo. The PELDOR signal was recorded with a shot repetition time of 3 ms. To achieve an acceptable SNR, the signal was averaged for 4 to 24 h, depending on the position of the detection pulses.

### 3.5. Stability Measurements

#### 3.5.1. Sample Preparation for Measurements with Ascorbic Acid

A 5 mM solution of L-ascorbic acid (Sigma–Aldrich) was prepared in PBS solution. Next, 1 µL spin label stock solution (4 mM) was mixed with 19 µL l-ascorbic acid solution at the time t_0_. The sample solution (200 µM spin label, 4.75 mM l-ascorbate) was drawn into a 10 µL capillary and sealed on both ends with Korasilon-Paste (Kurt Obermeier GmbH & Co., KG). The capillary was inserted into a capped Q-band tube.

#### 3.5.2. HeLa Cell Lysis Preparation

The HeLa cell pellet (2.13 × 10^8^ HeLa S3 cells, *ATCC# CCL-2.2*) was thawed on ice and resuspended in 2.13 mL PBS solution. The suspension was frozen in a dry ice/ethanol bath (−72 °C) for 5 min and then thawed in a warm water bath (37 °C) for 5 min. This cycle of freezing and thawing was repeated twice for a total of three circulations. Next, the suspension was rigorously mixed by vortexen and centrifuged at 15,000 rotations per minute (4 °C). The supernatant was collected and separated into aliquots with a volume of 19 µL each. The aliquots were shock frozen in liquid nitrogen and stored at −80 °C.

#### 3.5.3. Sample Preparation for Measurements in HeLa Lysate

One aliquot of HeLa lysate was thawed on ice. Next, 1 µL spin label stock solution (4 mM) was mixed with 19 µL lysate at the time t_0_. The sample solution (200 µM spin label) was drawn into a 10 µL capillary and sealed on both ends with Korasilon-Paste (Kurt Obermeier GmbH & Co., KG). The capillary was inserted into a capped Q-band tube.

#### 3.5.4. EPR Stability Measurements

All EPR spectra were measured in the EMX microX (Bruker BioSpin, Rheinstetten, Germany) using a SHQ resonator. The apparatus was controlled with the Bruker software Xenon. Before each measurement, the spectrometer was allowed to heat up for at least one hour to assure optimal magnetic field stability.

The SHQ resonator was tuned to the sample and the Q-value was taken at a 33 dB attenuation. The measurement was started at the time t_start_. The dead time was calculated as the difference between t0 and tstart. All samples were measured in a field-delay experiment for 21 h with one measurement slice every five minutes at a power attenuation of 15 dB and 30 points/G resolution. The experiments were done at room temperature with a modulation amplitude of 1 G. The tuning parameters—lock offset, diode current, microwave frequency, and receiver level—were observed using a home written shell program.

#### 3.5.5. Data Analysis

The raw spectrum was displayed in the Bruker software Xenon (Bruker BioSpin, Rheinstetten, Germany) and baseline corrected using a ninth order polynomial. The double integrals were calculated with the same software. The data were analyzed using a home written MATLAB script.

## 4. Conclusions

Spin label **2**^•^ was easily conjugated to a 16 nucleotide long self-complementary RNA strand through “click”-chemistry. The influence of the label on the duplex structure was analyzed with CD spectroscopy and thermal denaturation experiments and judged as being local and comparable to the previously published data on the *gem*-dimethyl analogue **1**^•^. This is also supported by the analysis of the PELDOR data, showing that both labels adopted very similar conformations on the duplex. Thus, the larger *gem*-diethyl substituents did not influence the alignment of spin label **2**^•^ in the RNA structure. Interestingly, the RNA duplex with spin labels **1**^•^ or **2**^•^ revealed a prolonged *T_M_* relaxation time that was not reported for similar isoindoline spin-labeled proteins [46], making longer distance measurements up to 10 nm feasible. Finally, spin label **2**^•^ had a considerably improved reduction stability against ascorbate and HeLa cell lysate as compared to MTSL. Therefore, **2**^•^ appears to be a promising candidate for long-range distance measurements on oligonucleotides within cells.

## Figures and Tables

**Table 1:** Spin echo decay data of **A_2_** and **B_2_** extracted from Q-Band two-pulse ESEEM.

Parameter	A_2_		B_2_	
	D_2_O	D_2_O + 17% H_2_O	D_2_O	D_2_O + 17% H_2_O
$Y_{0}$	0.56	0.57	0.47	0.42
$T_{M1}$/µs	20.7	13.4	21.1	13.7
x1	1.9	1.8	2.0	2.2
$T_{M2}$/µs	1.0	1.0	1.0	1.0
x2	0.8	0.8	0.8	0.8

**Table 2:** Geometric parameters of the PeldorFit analysis for **A_2_** and **B_2_**.

Parameter			A_2_		B_2_	
*r*	Δr	(nm)	4.74 (0.06) *	0.22 (0.07)	4.80 (0.06)	0.22 (0.08)
ξ	Δξ	(°)	37/143 (10)	7 (10)	44/136 (10)	4 (15)
β	Δβ	(°)	56/124 (5)	25 (15)	34/146 (5)	10 (15)

* The value in round brackets is the error of this parameter. The error is the parameter range in which 110% of the minimal RMSD is reached.

## Supplementary Materials

The Supplementary Materials are available online. It contains additional figures, spectra and details of the experiments and methods [47,58,59,60,61,62,63,64,65].

## References

[1] Karijolich J., Yu Y.-T. (2010). Spliceosomal snRNA modifications and their function. RNA Biol..

[2] Alberts B., Johnson A., Lewis J., Morgan D., Raff M., Roberts K., Walter P. (2014). Molecular Biology of the Cell.

[3] Lilley D.M.J., Eckstein F. (2008). Ribozymes and RNA Catalysis.

[4] Breaker R.R. (2018). Riboswitches and Translational Control. CSH Perspect. Biol..

[5] Reyes F.E., Garst A.D., Batey R.T. (2009). Strategies in RNA Crystallography. Methods in Enzymology.

[6] Zheng L., Falschlunger C., Huang K., Mairhofer E., Yuan S., Wang J., Patel D.J., Micura R., Rem A. (2019). Hatchet ribozyme structure and implications for cleavage mechanism. Proc. Natl. Acad. Sci. USA.

[7] Herbert H. (2019). CryoEM: A crystals to single particles round-trip. Curr. Opin. Struct. Biol..

[8] Kaledhonkar S., Fu Z., Caban K., Li W., Chen B., Sun M., Gonzalez R.L., Frank J. (2019). Late steps in bacterial translation initiation vizualized using time-resolved cryo-EM. Nature.

[9] Thompson R.D., Baisden J.T., Zhang Q. (2019). NMR Characterization of RNA Small Molecule Interactions. Methods.

[10] Garcia-Lopez A., Tessaro F., Jonker H.R.A., Wacker A., Richter C., Comte A., Berntenis N., Schmucki R., Hatje K., Petermann O. (2018). Targeting RNA structure in SMN2 reverses spinal muscular atrophy molecular phenotypes. Nat. Commun..

[11] Goldfarb D., Stoll S. (2018). EPR Spectroscopy: Fundamentals and Methods.

[12] Hetzke T., Vogel M., Gophane D.B., Weigand J.E., Suess B., Sigurdsson S.T., Prisner T.F. (2018). Influence of Mg^2+^ on the conformational flexibility of a tetracycline aptamer. RNA.

[13] Lerner E., Corder T., Ingargiola A., Alhadid Y., Chung S.Y., Michalet X., Weiss S. (2018). Toward dynamic structural biology: Two decades of single-molecule Förster resonance energy transfer. Science.

[14] Andreo A.Z., Harm U., Klostermeier D. (2019). Single-stranded regions modulate conformational dynamics and ATPase activity of elF4A to optimize 5′-UTR unwinding. Nucleic Acids Res..

[15] Chen Y., Pollack L. (2016). SAXS Studies of RNA: Structures, dynamics, and interactions with partners. WIRES RNA.

[16] Šponer J., Bussi G., Krepl M., Banáš P., Bottaro S., Cunha R.A., Gil-Ley A., Pinamonti G., Poblete S., Jurečka P. (2018). RNA Structural Dynamics As Captured by Molecular Simulations: A Comprehensive Overview. Chem. Rev..

[17] Hunsicker-Wang L., Vogt M., DeRose V.J. (2009). EPR Methods to Study Specific Metal-Ion Binding Sites in RNA. Meth. Enzymol..

[18] Kisseleva N., Kraut S., Jäschke A., Schiemann O. (2007). Characterizing multiple metal ion binding sites within a ribozyme by cadmium induced EPR silencing. HFSP J..

[19] Shelke S.A., Sigurdsson S.T., Timmel C., Harmer C., Anderson J. (2011). Structural Information from Spin-Labels and Intrinsic Paramagnetic Centres in the Biosciences. Structure and Bonding.

[20] Haugland M.M., Lovett J.E., Anderson E.A. (2018). Advances in the synthesis of nitroxide radicals for use in biomolecule spin labelling. Chem. Soc. Rev..

[21] Shevelev G.Y., Krumkacheva O.A., Lomzov A.A., Kuzhelev A.A., Rogozhinkova O.Y., Trukhin D.V., Troitykaya T.I., Tormyshev V.M., Fedin M.V., Pyshnyi D.V. (2014). Physiological-Temperature Distance Measurement in Nucleic Acid using Triarylmethyl-Based Spin Labels and Pulsed Dipolar EPR Spectroscopy. J. Am. Chem. Soc..

[22] Yang Z., Li Y., Borbat P., Zweier J.L., Freed J.H., Hubbell W.L. (2012). Pulsed ESR Dipolar Spectroscopy for Distance Measurements in Immobilized Spin Labeled Proteins in Liquid Solution. J. Am. Chem. Soc..

[23] Reginsson G.W., Kunjir N.C., Sigurdsson S.T., Schiemann O. (2012). Trityl Radicals: Spin Labels for Nanometer-Distance Measurements. Chem. Eur. J..

[24] Goldfarb D. (2014). Gd^3+^ spin labeling for distance measurements by pulse EPR spectroscopy. Phys. Chem. Chem. Phys..

[25] Wojciechowski F., Groß A., Holder I.T., Knörr L., Drescher M., Hartig J.S. (2015). Pulsed EPR spectroscopy distance measurements of DNA internally labelled with Gd^3+^-DOTA. Chem. Commun..

[26] Schiemann O., Weber A., Edwards T.E., Prisner T.F., Sigurdsson S.T. (2003). Nanometer Distance Measurements on RNA Using PELDOR. J. Am. Chem. Soc..

[27] Saha S., Jagtap A.P., Sigurdsson S.T. (2015). Site-directed spin labeling of 2′-amino groups in RNA with isoindoline nitroxides that are resistant to reduction. Chem. Commun..

[28] Qin P.Z., Butcher S.E., Feigon J., Hubbell W.L. (2001). Quantitative Analysis of the Isolated GAAA Tetraloop/Receptor Interaction in Solution: A Site-Directed Spin Labeling Study. Biochemistry.

[29] Nguyen P.H., Popova A.M., Hidgen K., Qin P.Z. (2015). A nucleotide-independent cyclic nitroxide label for monitoring segmental motions in nucleic acids. BMC Biophys..

[30] Esquiaqui J.M., Sherman E.M., Ionescu S.A., Ye J.-D., Fanucci G.E. (2014). characterizating the Dynamics of the Leader-Linker Interaction in the Glycine Riboswitch with Site-Directed Spin Labeling. Biochemistry.

[31] Sicoli G., Wachowius F., Bennati M., Höbartner C. (2010). Probing Secondary Structures of Spin-Labeled RNA by Pulsed EPR Spectroscopy. Angew. Chem. Int. Ed..

[32] Schiemann K., Piton N., Plackmeyer J., Bode B.E., Prisner T.F., Engels J.W. (2007). Spin labeling of oligonucleotides with the nitroxide TPA and use of PELDOR, a pulse EPR method, to measure intramolecular distances. Nat. Protoc..

[33] Höbartner C., Sicoli G., Wachowius F., Gophane D.B., Sigurdsson S.T. (2012). Synthesis and Characterization of RNA Containing a Rigid and Nonperturbing Cytidine-Derived Spin Label. J. Org. Chem..

[34] Kerzhner M., Abdullin D., Więcek J., Matsuoka H., Hagelueken G., Schiemann O., Famulok M. (2016). Post-synthetic Spin Labeling of RNA through Click Chemistry for PELDOR Measurements. Chem. Eur. J..

[35] Weinreich T., Jaumann E.A., Scheffer U., Prisner T.F., Göbel M.W. (2018). A Cytidine Phosporamidite with Protected nitroxide Spin Label: Synthesis of a Full-Length TAR RNA and Investigation by In-Line Probing and EPR Spectroscopy. Chem. Eur. J..

[36] Juliusson H.Y., Segler A.-L.J., Sigurdsson S.T. (2019). Benzoyl-Protected Hydroxylamines for Improved Chemical Synthesis of Oligonucleotides Containing Nitroxides Spin Labels. Eur. J. Org. Chem..

[37] Schmidt T., Wälti M.A., Baber J.L., Hustedt E.J., Clore G.M. (2016). Long Distance Measurements up to 160 Å in the GroEL Tetradecamer Using Q-Band DEER EPR Spectroscopy. Angew. Chem. Int. Ed..

[38] Kristić I., Endeward B., Margraf D., Marko A., Prisner T.F., Klostermeier D., Hamman D. (2012). EPR Spectroscopy Applications in Chemistry and Biology.

[39] Schiemann O. (2009). Mapping Global Folds in Oligonulceotides by Pulsed Electron-electron Double Resonance. Methods in Enzymology.

[40] Marx L., Chiarelli R., Guiberteau T., Rassat A. (2000). A comparative study of the reduction by ascorbate of 1,1,3,3-tetraethylisoindolin-2-yloxyl and of 1,1,3,3-tetramethylisoindolin-2-yloxyl. J. Chem. Soc. Perkin Trans..

[41] Paletta J.T., Pink M., Foley B., Rajca S., Rajca A. (2012). Synthesis and Reduction Kinetics of Sterically Shielded Pyrrolidine Nitroxides. Org. Lett..

[42] Wang Y., Paletta J.T., Berg K., Reinhart E., Rajca S., Rajca A. (2014). Synthesis of Unnatural Amino Acids Functionalized with sterically Shielded Pyrroline Nitroxides. Org. Lett..

[43] Jagtap A.P., Krstic I., Kunjir N.C., Hänsel R., Prisner T.F., Sigurdsson S.T. (2015). Sterically shielded spin labels for in-cell EPR spectroscopy: Analysis of stability in reducing environment. Free Radic. Res..

[44] Karthikeyan G., Bonucci A., Casano G., Gerbaud G., Abel S., Thomé V., Kodjabachian L., Magalon A., Guigliarelli B., Belle V. (2018). A Bioresistant Nitroxide Spin Label for In-Cell EPR Spectroscopy: In Vitro and In Oocytes Protein Structural Dynamics Studies. Angew. Chem. Int. Ed..

[45] Bleicken S., Assafa T.E., Zhang H., Elsner C., Ritsch I., Pink M., Rajca S., Jeschke G., Rajca A., Bordignon E. (2019). gem-Diethyl Pyrroline Nitroxide Spin Labels: Synthesis, EPR Characterization, Rotamer Libraries and Biocompatibility. Chemistryopen.

[46] Braun T.S., Widder P., Osswald U., Groß L., Williams L., Schmidt M., Helmle I., Summerer D., Drescher M. (2019). Isoindoline-Base Nitroxides as Bioresistant Spin Labels for Protein Labeling via Cysteines and Alkyne Bearing Noncanonical Amino Acids. ChemBioChem.

[47] Haugland M.M., El-Sagheer A.H., Porter R.J., Peña J., Brown T., Anderson E.A., Lovett J.E. (2016). 2′-Alkynylnucleotides: A Sequence- and Spin Label-Flexible Strategy for EPR Spectroscopy in DNA. J. Am. Chem. Soc..

[48] Kerzhner M., Matsuoka H., Wuebben C., Famulok M., Schiemann O. (2018). High-Yield Spin Labeling of Long RNAs for Electron Paramagnetic Resonance Spectroscopy. Biochemistry.

[49] Sproules S., Chechik V., Murphy D.M. (2017). Molecules as electron spin qubits. Electron Paramagnetic Resononance.

[50] Eaton S.S., Eaton G.R., Berliner L.J., Eaton G.R., Eaton S.S. (2000). Distance Measurements in Biological Systems by EPR.

[51] Lindgren M., Eaton G.R., Eaton S.S., Jonsson B.-H., Hammarström P., Svensson M., Carlsson U. (1997). Electron spin echo decay as a probe of aminoxyl environment in spin-labeled mutants of human carbonic anhydrase II. J. Chem. Soc. Perkin Trans..

[52] Huber M., Lindgren M., Hammarström P., Mårtensson L.-G., Carlsson U., Eaton G.R., Eaton S.S. (2001). Phase memory relaxation times of spin labels in human carbonic anhydrase II: Pulsed EPR to determine spin label location. Biophys. Chem..

[53] Ward R., Bowman A., Sozudogru E., El-Mkami H., Owen-Hughes T., Norman D.G. (2010). EPR Distance measurements in deuterated proteins. J. Magn. Reson..

[54] El-Mkami H., Ward R., Bowman A., Owen-Hughes T., Norman D.G. (2014). The spatial effect of protein deuteration on nitroxide spin-label relaxation: Implication for EPR distance measurement. J. Magn. Reson..

[55] Zecevic A.A., Eaton G.R., Eaton S.S., Lindgren M. (1998). Dephasing of electron spin echoes for nitroxyl radicals in glassy solvents by non-methyl and methyl protons. Mol. Phys..

[56] Jeschke G., Polyhach Y. (2007). Distance measurements on spin-labelled biomacromolecules by pulsed electron paramagnetic resonance. Phys. Chem. Chem. Phys..

[57] Kulik L.V., Dzuba S.A., Grigoryev I.A., Tsvetkov Y.D. (2001). Electron dipole-dipole interaction in ESEEM of nitroxide biradicals. Chem. Phys. Lett..

[58] Abdullin D., Hagelueken G., Hunter R.I., Smith G.M., Schiemann O. (2014). Geometric model-based fitting algorithm for orientation-selective PELDOR data. Mol. Phys..

[59] Goddard-Boger E.D., Stick R.V. (2007). An Efficient, Inexpensive, and Shelf-Stable Diazotransfer Reagent: Imidazole-1-sulfonyl Azide Hydrochloride. Org. Lett..

[60] Stoll S., Schweiger A. (2006). EasySpin, a comprehensive software package for spectral simulation and analysis in EPR. J. Magn. Reson..

[61] Gafurov M., Lyubenova S., Denysenkov V., Ouari O., Karoui H., Le Moigne F., Tordo P., Prisner T. (2009). EPR Charaterization of a Rigid Bis-TEMPO-Bis-Ketal for Dynamic Nuclear Polarization. App. Magn. Reson..

[62] Windle J.J. (1981). Hyperfine Coupling Constantes for Nitroxide Spin Probes in Water and Carbon Tetrachloride. J. Magn. Reson..

[63] Hagelueken G., Ward R., Naismith J.H., Schiemann O. (2012). MtsslWizard: In Silico Spin-Labeling and Generation of Distance Distributions in PyMOL. Appl. Magn. Reson..

[64] Hagelueken G., Abdullin D., Ward R., Schiemann O. (2013). mtsslSuite: In silico spin labelling, trilateration and distance-constrained rigid body docking. Mol. Phys..

[65] Hagelueken G., Abdullin D., Schiemann O. (2015). mtsslSuite: Probing Biomolecular Conformation by Spin-Labeling Studies. Methods in Enzymology.

