# Supplementary material for: Site-Directed Spin Labeling of RNA with a Gem-Diethylisoindoline Spin Label: PELDOR, Relaxation, and Reduction Stability
Source: Molecules. 2019 Dec 6;24(24):4482. doi: 10.3390/molecules24244482 (PMC6943706; doi:10.3390/molecules24244482)
Supplement: Supplementary file 1 [file molecules-24-04482-s001.pdf]

molecules

Site-Directed Spin Labeling of RNA with A *Gem*-Diethylisoindoline Spin Label: PELDOR, Relaxation, and Reduction Stability

Christine Wuebben [1], Simon Blume [1], Dinar Abdullin [1], Dominik Brajtenbach [1], Florian Haege [1], Stephanie Kath-Schorr [2] and Olav Schiemann [1,*]

[1] Institute of Physical and Theoretical Chemistry, University of Bonn, Wegelerstr. 12, 53115 Bonn, Germany; wuebben@pc.uni-bonn.de (C.W.); s6siblum@uni-bonn.de (S.B.); abdullin@pc.uni-bonn.de (D.A.); s6dobraj@uni-bonn.de (D.B.); Florian-Haege@web.de (F.H.)

[2] Life & Medical Sciences Institute Chemical Biology & Medicinal Chemistry Unit, University of Bonn, Gerhard-Domagk-Str. 1, 53121 Bonn, Germany; stephanie.kath-schorr@uni-bonn.de

* Correspondence: schiemann@pc.uni-bonn.de

Supporting Information

Contents

1. Synthesis and Analytics of 2• ... 3
2. cw EPR ... 3
3. 2-Pulse ESEEM Measurements ... 4
4. PELDOR Measurements ... 5
5. PELDOR Data Analysis ... 6
6. Stability Measurements under Reducing Conditions ... 9
   6.1. DNA Sequence ... 9
   6.2. Spin Labeling Reaction ... 9
   6.3. DNA Sample Preparation ... 9

1. Synthesis and Analytics of 2•

The azide functionalized *gem*-diethylisoindoline spin label **2•** (Figure 1b) was synthesized in six steps starting from N-benzylphtalimid with slight modification according to the synthesis reported by Haugland et al. [47]. In order to avoid handling the explosive diazotransfer reagent trifluoromethanesulfonyl azide, imidazole-1-sulfonyl azide hydrochloride was used following the protocol of Goddard-Borger et al. [59]. This led to a yield of 70% for the diazotransfer reaction as compared to the reported 87% for the trifluoromethanesulfonyl azide reaction [47]. Spin label **2•** was obtained as a yellow powder in an overall yield of 7%. Its identity and purity were confirmed by high-performance liquid chromatography, IR and EPR spectroscopy, as well as mass spectrometry (Figure S1). In the experimental high-resolution ESI (+) mass spectrum, a negligible amount of a species at 289.2023 m/z was detectable (Figure S1a), which was assigned to the corresponding hydroxylamine (Figure S1c) formed during the ESI measurement, because there was no additional peak in the HPLC analysis.

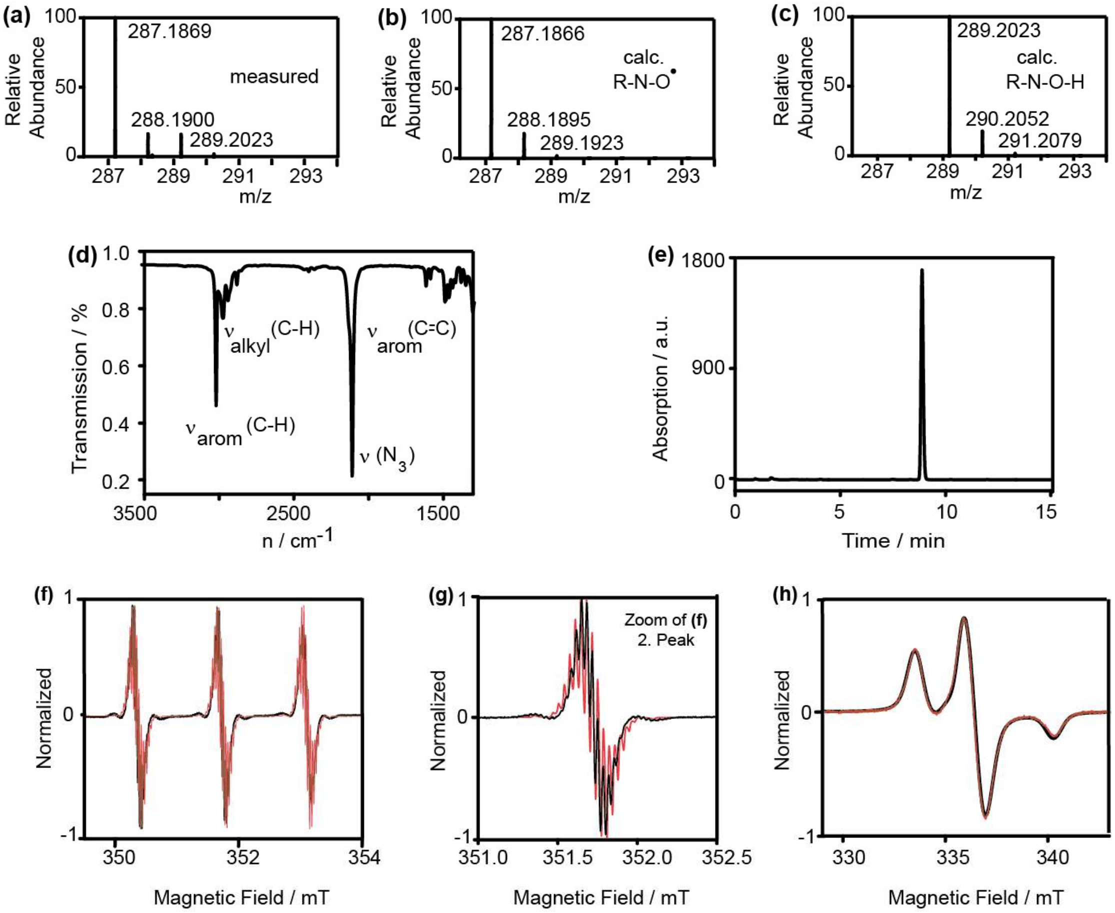

Figure S1. Analytics of spin label **2•**. (**a**) Experimental high-resolution ESI (+) mass spectrum. (**b**) Calculated high-resolution ESI (+) mass spectrum of spin label **2•**. (**c**) Calculated high-resolution ESI (+) mass spectrum of the corresponding hydroxylamine. (**d**) IR spectrum. (**e**) HPLC run. (**f**) Experimental cw X-band EPR spectrum in liquid toluene at 295 K (black line) overlaid with the simulation (red line). (**g**) Zoom in for the 2nd Peak in (**f**) and (**h**) experimental cw X-band EPR spectrum in acetonitrile: methylene chloride 1:1 at 100 K (black line) overlaid with the simulation (red line).

2. cw EPR

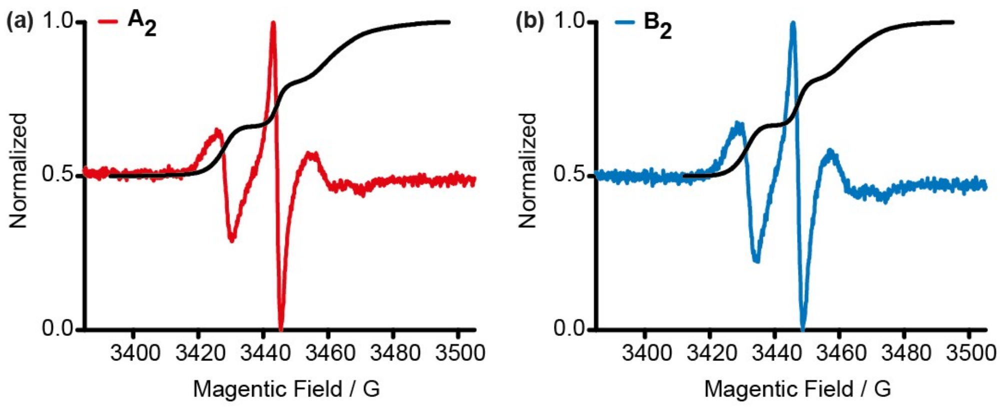

Figure S2. Experimental cw EPR spectrum of (**a**) **A**2 and (**b**) **B**2 overlaid with their double integral.

Table S1. Spin counting results of **A**2 and **B**2.

Spin Counting	Concentration of the RNA/µM	Calculated Spin Concentration/µM
**A**2	35	32
**B**2	35	31

Table S2. Parameters of the cw EPR simulations using easySpin [3] of spin label 2•.

Parameter	2• 295 K	Nitroxide-Biradical 295 K [61] [a]	Tempo 295 K [62] [b]	2• 100 K [c]
g-tensor	2.0036 [d]	2.0063		2.0092, 2.0062, 2.0029 [e]
A-tensor/MHz	38, 9, 1 [f]	42	44, 0.6/1, 0.5 [f]	12, 12, 94 [g]

[a] Experimental values of a Bis-TEMPO-bis-Ketal in d8-toluene. [b] Experimental values of TEMPO in CCl_4. [c] For the simulation in frozen solution, an H-strain of 142,420 MHz was used. [d] Isotropic g-value. [e] g-Tensor is given in the form (g_{xx}, g_{yy}, g_{zz}). [f] The hyperfine coupling constants in liquid solution are given in the form ($A_{iso}(^{14}N)$, $A_{iso}(^{13}C)$, $A_{iso}(^{1}H)$). [g] The $A(^{14}N)$ tensor at 100 K is given in the form (A_{xx}, A_{yy}, A_{zz}).

3. 2-Pulse ESEEM Measurements

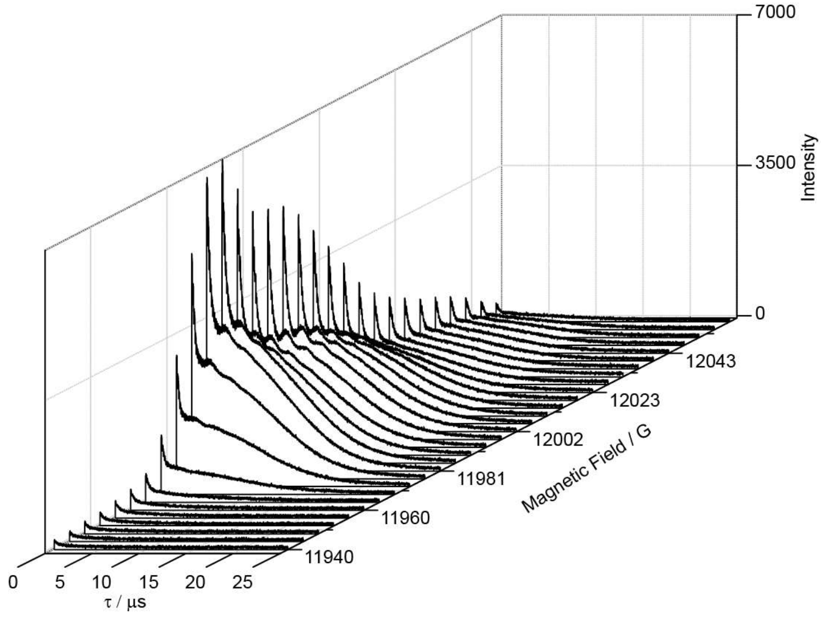

Figure S3. Experimental two-pulse ESEEM spectra of **B**$_2$ at 50 K in dependence of the magnetic field.

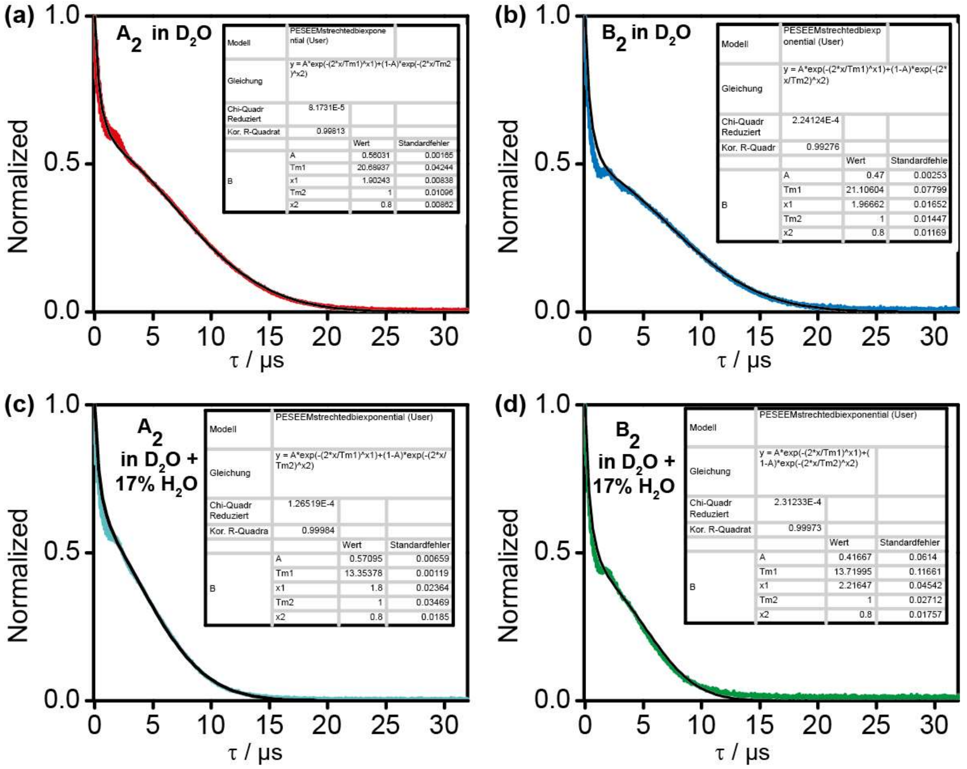

Figure S4. Experimental two-pulse ESEEM spectra recorded at 50 K overlaid with their corresponding fits (black line): (**a**) **A**$_2$ in deuterated phosphate buffer (red line), (**b**) **B**$_2$ in deuterated phosphate buffer (blue line), (**c**) **A**$_2$ in deuterated phosphate buffer with additional 17% water (light blue line), (**d**) **B**$_2$ in deuterated phosphate buffer with additional 17% water (green line).

4. PELDOR Measurements

Table S3. Frequency offsets.

Notation	Pump Position	Detection Position	Frequency Offset/MHz
$\Delta\nu_{ax}$	a	x	40
$\Delta\nu_{bx}$	b	x	40
$\Delta\nu_{ab}$	a	b	80
$\Delta\nu_{be}$	b	e	80
$\Delta\nu_{ac}$	a	c	100
$\Delta\nu_{ae}$	a	e	160

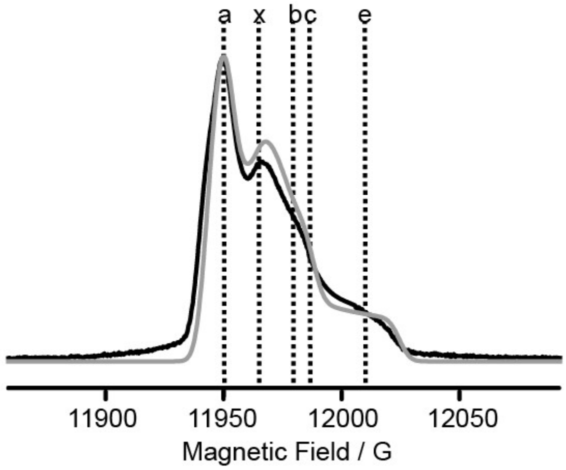

Figure S5. Experimental echo detected field sweep in Q-band of **B**2 (black line) overlaid with the Simulation (grey line). In dashed lines and depicted in letters are the different pump and detection positions marked.

Table S3. Frequency offsets.

Notation	Pump Position	Detection Position	Frequency Offset/MHz
$\Delta\nu_{ax}$	a	x	40
$\Delta\nu_{bx}$	b	x	40
$\Delta\nu_{ab}$	a	b	80
$\Delta\nu_{be}$	b	e	80
$\Delta\nu_{ac}$	a	c	100
$\Delta\nu_{ae}$	a	e	160

Table S4. Parameters used for simulation of the field sweep spectrum in Figure S5.

Parameter	50 K
g-tensor	2.0086, 2.0064, 2.0026
A-tensor/MHz	18, 18, 102
Line width/MHz	19.6

5. PELDOR Data Analysis

The PeldorFit program [58], which is based on

$$\nu_{dd} = \frac{g_A g_B \mu_B^2 \mu_0}{4\pi\hbar} \times \frac{1}{r^3}(1 - 3\cos^2\theta) \quad (1)$$

was used to analyze the orientation selectivity. The configuration file contains three main blocks of information filled in by the user:

1. Instrumental parameters of the PELDOR experiment;

2. Spectroscopic parameters of the involved spins which were obtained by simulating the experimental Q-band spectrum with easySpin [60] (Figure S5). The parameter from the fit are collected in Table S4;

3. The parameters of Table S5 were used as fitting parameters assuming rhombic magnetic tensors for the spins.

The program fits the orientation selective time traces by means of a genetic algorithm and yields the geometric parameters r, ξ, φ, α, β, and γ of a simplified geometric model (Figure

S6) and their distributions. The genetic algorithm was set to a maximal number of generations of 200 and a generation size of 192. The geometric parameters are optimized within the ranges given in Table S5 until the corresponding RSMD reached a minimum. The results are given in Table S6 and S7 including the 16 symmetry-related sets of parameters due to the invariance of the g- and A-tensor towards inversion of their axes.

Table S5. Fitting parameters for PeldorFit.

Parameter	Mean	Width
r/nm	4–5	0–1
ξ/°	0–90	0–30
φ/°	0–180	0–60
α/°	0–180	0–60
β/°	0–90	0–30
γ/°	0–180	0–60

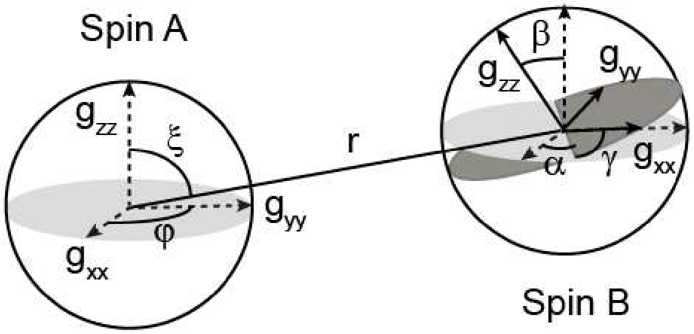

Figure S6. Model of the geometric parameters for PeldorFit.

Table S6. Summary of all sets of angles for **A$_2$** of the analysis of the PELDOR time traces using the PeldorFit program.

**Transformation ***	**ξ/°**	**φ/°**	**α/°**	**β/°**	**γ/°**	**RMSD**
Fitting result	37	16	154	56	150	0.038
Inversion of gxxA	37	16	334	124	30	0.038
Inversion of gyyA	37	16	334	124	210	0.037
Inversion of gzzA	37	16	154	56	330	0.038
Inversion of gxxB	143	344	26	124	330	0.038
Inversion of gyyB	143	344	206	56	210	0.038
Inversion of gzzB	143	344	206	56	30	0.037
Inversion of gxxA and gxxB	143	344	26	124	150	0.037
Inversion of gxxA and gyyB	143	164	206	124	330	0.037
Inversion of gxxA and gzzB	143	164	26	56	210	0.038
Inversion of gyyA and gxxB	143	164	26	56	30	0.037
Inversion of gyyA and gyyB	143	164	206	124	150	0.037
Inversion of gyyA and gzzB	37	196	334	56	150	0.037
Inversion of gzzA and gxxB	37	196	154	124	30	0.037
Inversion of gzzA and gyyB	37	196	154	124	210	0.037
Inversion of gzzA and gzzB	37	196	334	56	330	0.038

* g_{xx}^A, g_{yy}^A, and g_{zz}^A denote the principal components of the g-tensor of spin A; g_{xx}^B, g_{yy}^B, and g_{zz}^B denote the g-tensor of spin B.

Table S7. Summary of all sets of angles for B$_2$ of the analysis of the PELDOR time traces using the PeldorFit program.

**Transformation ***	**ξ/°**	**φ/°**	**α/°**	**β/°**	**γ/°**	**RMSD**
Fitting result	44	155	175	34	19	0.048
Inversion of gxxA	44	155	355	146	161	0.048
Inversion of gyyA	44	155	355	146	341	0.048
Inversion of gzzA	44	155	175	34	199	0.048
Inversion of gxxB	136	205	5	146	199	0.048
Inversion of gyyB	136	205	185	34	341	0.048
Inversion of gzzB	136	205	185	34	161	0.048
Inversion of gxxA and gxxB	136	205	5	146	19	0.048
Inversion of gxxA and gyyB	136	25	185	146	199	0.048
Inversion of gxxA and gzzB	136	25	5	34	341	0.048
Inversion of gyyA and gxxB	136	25	5	34	161	0.048
Inversion of gyyA and gyyB	136	25	185	146	19	0.048
Inversion of gyyA and gzzB	44	335	355	34	19	0.048
Inversion of gzzA and gxxB	44	335	175	146	161	0.048
Inversion of gzzA and gyyB	44	335	175	146	341	0.048
Inversion of gzzA and gzzB	44	335	355	34	199	0.048

* g_{xx}^A, g_{yy}^A, and g_{zz}^A denote the principal components of the g-tensor of spin A; g_{xx}^B, g_{yy}^B, and g_{zz}^B denote the g-tensor of spin B.

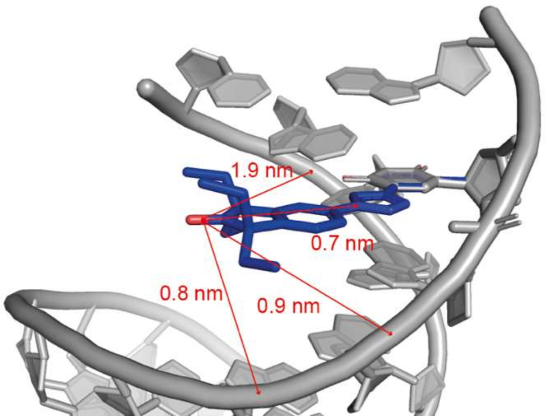

Figure S7. Pymol illustration of the critical range of protons in the vicinity of the spin labels labeled within MtsslWizard [63–65].

6. Stability Measurements under Reducing Conditions

6.1. DNA Sequence

The DNA strands 5′ GGG TGX CTG GTA CCC 3′ and 5′ A GGG TAC CAG ACA CCC A 3′ were purchased from metabion.

6.2. Spin Labeling Reaction

The spin labeling was conducted in the same way as the labeling of the RNA (see Section 3.1.2.). Afterwards, the DNA was purified through reverse-phase high-performance liquid chromatography with an Agilent 1200 Series HPLC System (Agilent Technology, Santa Clara, CA, USA) in combination with a Zorbax 300SB-C18 (4.6 mm × 150 mm) column (Agilent Technologies, Santa Clara, CA, USA). As the eluent was a 0.1 M aqueous solution of triethylammonium acetate (VWR Applichem), an increasing percentage of acetonitrile (VWR Chemicals) (8% to 20% over 14 min, then up to 80% acetonitrile for 15 min) was used.

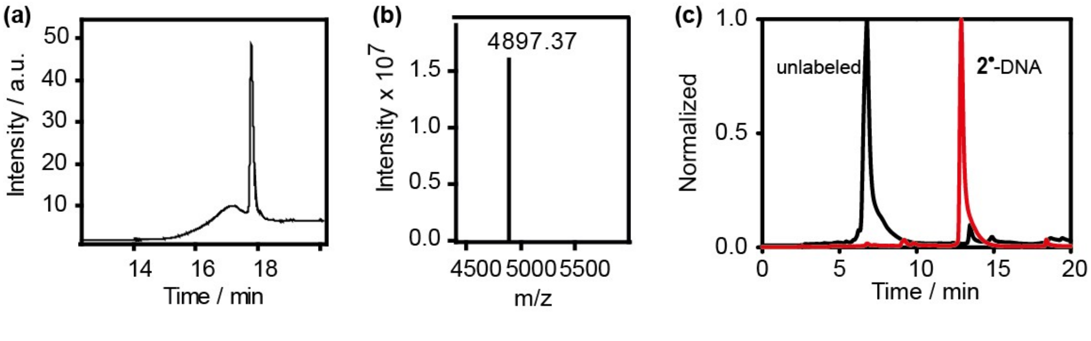

Figure S8. (**a**) Chromatogram at 260 nm and (**b**) deconvoluted mass of **2$^•$** DNA (calculated mass 4897.925, found mass 4897.37). (**c**) Overlay of the HPLC runs of the unlabeled (black line) and labeled DNA (red line).

6.3. DNA Sample Preparation

The labeled single DNA strand was mixed 1:1 with the unmodified DNA strand in phosphate-buffered saline solution (PBS; 137 mM sodium chloride (Carl Roth), 10 mM sodium hydrogen phosphate (Carl Roth), 2.7 mM potassium chloride (Carl Roth), 1.8 mM potassium dihydrogen

phosphate (Carl Roth) pH 7.4). The mixture was denaturated for 5 min at 70 °C and incubated for at least 15 min at room temperature.

Table S8. Power settings, Q-value and time laps before EPR measurement.

	**Power / mW**	**Dead Time / min**	**Q-Value**
**2**$^{\bullet}$-DNA/Asc	5.529	11	6500
**2**$^{\bullet}$-DNA/HeLa	5.529	6	6200
MTSL/Asc	5.568	9	6500
MTSL/HeLa	5.537	5	6200
